# Evaluation of Factors Associated with Hypermetabolism and Hypometabolism in Critically Ill AKI Patients

**DOI:** 10.3390/nu10040505

**Published:** 2018-04-19

**Authors:** Cassiana R. de Góes, André Luis Balbi, Daniela Ponce

**Affiliations:** Internal Medicine Department, Faculdade de Medicina de Botucatu, São Paulo State University “UNESP—Univ Estadual Paulista”, Paranapanema Avenue, 165, Avaré, São Paulo 18701240, Brazil; abalbi@fmb.unesp.br (A.L.B.); dponce@fmb.unesp.br (D.P.)

**Keywords:** acute kidney injury, energy metabolism, dialysis, critical care

## Abstract

Acute kidney injury (AKI) is a frequent and serious condition with high mortality. The presence of hypermetabolism may be a factor related to poorer prognosis. This study evaluated the resting energy expenditure (REE) of intensive care unit (ICU) patients with severe AKI using indirect calorimetry (IC) and identified factors associated with metabolism categories. Patients were evaluated through measurement of REE and estimation of basal energy expenditure (BEE) using the Harris–Benedict equation. Metabolism categories were as follows: hypermetabolism (REE/BEE > 1.3) and hypometabolism (REE/BEE < 0.9). The metabolism categories were compared using ANOVA and the chi-square test. Variables were analyzed by multiple logistic regression tests. Also, survivors and non-survivors were compared using Student’s *t*-tests along with Cox regression tests. Kaplan–Meier survival curves were also performed. We evaluated 124 patients with a mean age of 61.08 ± 16.6 years. Sixty-four patients were hypermetabolic (62%) and 18 were hypometabolic (14%). Vasoactive drug (VAD) dose and younger age were independently associated with hypermetabolism. The survival analysis was not associated with metabolism categorization. In conclusion, patients with severe AKI are mostly hypermetabolic and hypermetabolic patients of a lower age receiving treatment with higher VAD doses. The only factors associated with death were protein intake and VAD dose.

## 1. Introduction

The development of acute kidney injury (AKI) is a frequent and serious condition in patients admitted to intensive care units (ICUs). Short-term mortality is high, reaching more than 50%, and is associated with AKI severity [1,2].

Usually, AKI forms part of more complex clinical conditions such as sepsis, multiple organ failure, shock, trauma, and high-risk surgeries, resulting in metabolism changes and catabolism. In addition, renal failure is also associated with major changes in substrate metabolism and body composition [3,4,5].

Although severe AKI is accompanied by metabolic changes and a catabolic process, AKI alone does not seem to increase energy expenditure (EE) significantly [6,7]. A study in the 1990s found the measured EE to be 30% higher in AKI patients when compared with the control group. Although this increase was only seen in patients with AKI associated with sepsis, the non-sepsis AKI group did not appear to have altered EE [7]. A more recent study from our group found the resting energy expenditure (REE) in patients with sepsis and AKI to be similar to that of patients with sepsis without AKI (1824.37 ± 751.74 kcal vs. 1909.12 ± 565.46 kcal, respectively, *p* = 0.63) [8].

Hypermetabolism and hypometabolism are observed in critically ill patients [9]. During clinical deterioration in septic shock, for example, EE decreases. On the other hand, during recovery from sepsis, EE can increase by more than 60% [10]. Thus, EE varies according to disease stage and may be a useful parameter with respect to clinical stage.

Hypermetabolism is a complication mediated by the immune system, which can be affected by damaged tissue rupture and/or pathogenic microorganisms and the entry of their toxins into the bloodstream, as well as the body’s response, for example hormone and cytokine release. Due to this situation, hypermetabolic patients often have higher mortality rates as compared to metabolically normal patients [9,11,12].

There is a gap in the literature about EE in patients with AKI, and to date, no study has evaluated the presence of hypermetabolism and clinical conditions that could be related to this situation in these patients. Thus, the present study aims to evaluate REE, as measured by indirect calorimetry (IC), in patients with severe AKI hospitalized in the ICU, and identify clinical factors associated with metabolism categorization. 

## 2. Materials and Methods

### 2.1. Subjects

The prospective cohort study evaluated patients aged over 18 years admitted to the ICU from March 2013 to December 2015 with a diagnosis of AKI stage 3 as per KDIGO (Kidney Disease: Improving Global Outcomes Group) 2012 criteria [13], with a clinical diagnosis of acute tubular necrosis (ATN) and an indication for renal replacement therapy. Patients were mechanically ventilated and IC was performed prior to the beginning of the dialysis procedure. Patients with AKI caused by other etiologies and stages 4 and 5 chronic kidney disease (glomerular filtration rate—GFR —<30 mL/min, as estimated by the Modification of Diet in Renal Disease eMDRD) [14] were excluded, as were kidney transplant patients. To estimate TFG, the patient’s baseline serum creatinine, defined as the most recent serum creatinine value obtained before admission (not preceding 12 months of the hospitalization date) was used. If this value was unknown, the lowest value observed during follow-up was considered [15].

Patients with AKI stage 1, 2, or 3 without dialysis, or those with an inspired fraction of oxygen (FiO_2_) greater than 0.60, positive end-expiratory pressure (PEEP) >10 cm H_2_O, maximum airway pressure >60 cm H_2_O, or leakage of air into the ventilator circuit, around the cuff of the endotracheal tube, or from a bronchopleural fistula, were excluded. Agitated patients or those being treated with neuromuscular blockers were also excluded. 

The protocol was started when the dialysis procedure was indicated, and the patient was followed up until hospital discharge due to recovery of renal function, or death. IC was performed on the day of dialysis indication, before the procedure was initiated.

This study was approved by the institutional ethics committee (protocol 4383/2012). The consent form was signed by the participant’s legal guardian prior to entry into the study.

### 2.2. Energy Expenditure Measure 

REE is usually measured due to the impossibility of achieving adequate conditions for measuring basal EE in critical patients. To ensure REE measurement, the patients were in supine position resting for at least 30 min prior to the measurement. The environment was thermoneutral for the 30 min prior to the measurement and during it. Use of additional painkillers or sedatives within 30 min of IC initiation was not permitted. No procedures were performed within 60 min of beginning the IC; no general anesthesia was administered within 8 h of IC onset; and parenteral and/or enteral nutrition were maintained in continuous infusion during the data collection period.

IC was performed using Quark RMR calorimeter (Cosmed, Rome, Italy), which was calibrated before each measurement. The exam lasted 30 min on average. It was desired that the patients should reach a steady state during the test. Steady state was defined as a variation of <10% in the oxygen consumption measurements (VO_2_) and carbon dioxide production (VCO_2_), and <5% in respiratory quotient each minute.

In addition to REE, variables that were potentially associated with EE were also evaluated, such as clinical parameters (body temperature, noradrenaline use, and presence of continuous sedation); laboratory tests (serum urea and creatinine, C-reactive protein—CRP, total lymphocyte count); ventilation parameters (minute volume, total current volume, PEEP, FIO_2_), dietary parameters (calorie and protein intake); and protein catabolism, which was calculated using urea nitrogen appearance (UNA) and nitrogen balance (NB) according to Drum [16].

### 2.3. Metabolism Categorization

There are several classifications for hyper/hypometabolism in the literature [9,12,17,18,19]. Since it has been described that patients with AKI and sepsis may have a REE approximately 30% higher than basal metabolism, and our population is mostly septic, we consider the definition of Brandi et al. to be more appropriate [12,17]. Thus, BEE was estimated by the Harris–Benedict formula [20] and hypermetabolism was considered to occur when the REE measured by IC was greater than 30% of the BEE. Hypometabolism was considered to occur when the measured REE was 10% lower than the predicted BEE [17].

Patient height (cm) was measured at ICU admission, when possible, or the value documented in the medical record was used. Weight (kg) was measured at admission, using calibrated hospital beds in the majority of patients. If the patient had edema at the time of the measurement, according to the medical evaluation, relatives were asked about the patient’s regular weight, and this value was used as the real weight in the formula to estimate BEE.

### 2.4. Statistical Analysis

Results are presented as the median and first and third quartiles, mean ± standard deviation, or as percentages. Different categories of metabolism were compared using ANOVA test, with post hoc Tukey or Kruskal–Wallis with post hoc Dunn and the chi-square test, according to distribution and normality characteristics. After the univariate test, the variables with significant differences between metabolism categories were analyzed with multiple logistic regression, using a forward selection method to consider which parameters were independently correlated with the presence of hypermetabolism or hypometabolism.

For the comparison between survivors and non-survivors, Student’s *t*-test or the Mann–Whitney test was used. After the univariate test, the variables with *p* < 0.10 were analyzed in Cox regression.

The collinearity of the predictive variables was tested using the tolerance and variance inflation factor (VIF), and if the tolerance was <0.1 and/or the VIF >4, then one of the variables was removed from the multivariate models.

Evaluation of difference in 28-day mortality of the three metabolic modalities was performed by using the Kaplan–Meier survival curve with log-rank test.

Statistical significance was considered as *p* < 0.05.

## 3. Results

One hundred twenty-four AKI patients in the ICU were evaluated at the time indication of the dialysis procedure. The mean age of the patients was 61.08 ± 16.6 years, and 69% were male. The etiology of AKI was associated with sepsis in most patients (80%). The Acute Tubular Necrosis Individual Score Severity (ATN-ISS) index-specific prognostic average was 0.65 ± 0.17. Regarding the REE measured using IC, the mean in the studied population was 2042 ± 703 kcal, a value significantly higher than the estimated BEE using the Harris–Benedict formula. Table 1 shows clinical characteristics of general study population.

According to REE classification, 64 patients were hypermetabolic (62%), 42 were normometabolic (34%), and 18 were hypometabolic (14%) at the time of indication for dialysis. Table 2 shows patient characteristics in different categories of EE. Patients of the different categories were similar with respect to gender, age, AKI etiology, main diagnosis, and outcome.

Differences in body weight were observed between groups. These differences were greater for hypometabolic patients (73.8 ± 19.7 kg in hypermetabolic group, 75.3 ± 16 kg in normometabolic group, and 96.2 ± 35.6 kg in hypometabolic group, *p* < 0.001). Similarly, hypometabolic patients had higher body mass index (BMI) than hypermetabolic and normometabolic patients. Groups also differed in body temperature (37.9 ± 1 °C in the hypermetabolic group, 37.5 ± 0.88 °C in the normometabolic group, and 37.6 ± 0.93 °C in the hypometabolic group, *p* = 0.04) and doses of vasoactive drug (noradrenaline), which were both higher in hypermetabolic patients as compared to normometabolic patients (0.28 (0.10–0.77) mcg/kg/min in hypermetabolic patients, 0.12 (0.02–0.23 mcg/kg/min) in normometabolic patients, and 0.15 (0.08–0.6) mcg/kg/min in hypometabolic patients, *p* = 0.02))

The groups also differed in terms of protein catabolism as evaluated by the UNA and NB. Hypermetabolic patients were less catabolic compared to normometabolic patients (11.2 (7.01, 15.3) g versus 18.44 (10.4, 23.9) g, respectively, *p* = 0.04; NB of −4.45 (−8.4, 1.88) g versus −8.4 (−17.8, −4.1) g, respectively, *p* = 0.02).

There was no difference in calorie intake between groups, while protein intake was significantly lower in the hypometabolic group.

A posteriori, we analyzed the variables that presented a statistically significant difference in univariate analysis for the occurrence of hypermetabolism and hypometabolism. The multiple logistic regression results are shown in Table 3 and Table 4. Age and VAD dose were independently associated with hypermetabolism and a marginal significance was observed for gender, indicating a possible benefit for males regarding the occurrence of hypermetabolism. Only body weight was independently associated with hypometabolism.

Seventy-three percent of the patients died within 28 days of the IC procedure. Patients who did not survive were older (53.73 ± 18.8 years versus 63.75 ± 15 years. *p* = 0.02), used higher VAD doses (0.08 (0–0.20) mcg/kg/min versus 0.23 (0.1–0.8) mcg/kg/min, *p* < 0.001), had higher body temperatures (37.4 ± 0.6 °C versus 37.8 ± 1 °C, *p* = 0.04), and lower serum creatinine (5.35 ± 2.8 mg/dL versus 4.10 ± 1.8 mg/dL, *p* = 0.004), as well as lower calorie and protein intake (840 (0–1536) versus 0 (0–1220) kcal and 64 (0–89.6) versus 0 (0–73) g, respectively, *p* = 0.03) when compared with survivors, at the time of the IC test. The characteristics of survivors and non-survivors are shown in Table 5.

After the multivariate analysis, the only variables that were independently associated with death were VAD dose and protein intake (Table 6).

**Figure 1:**
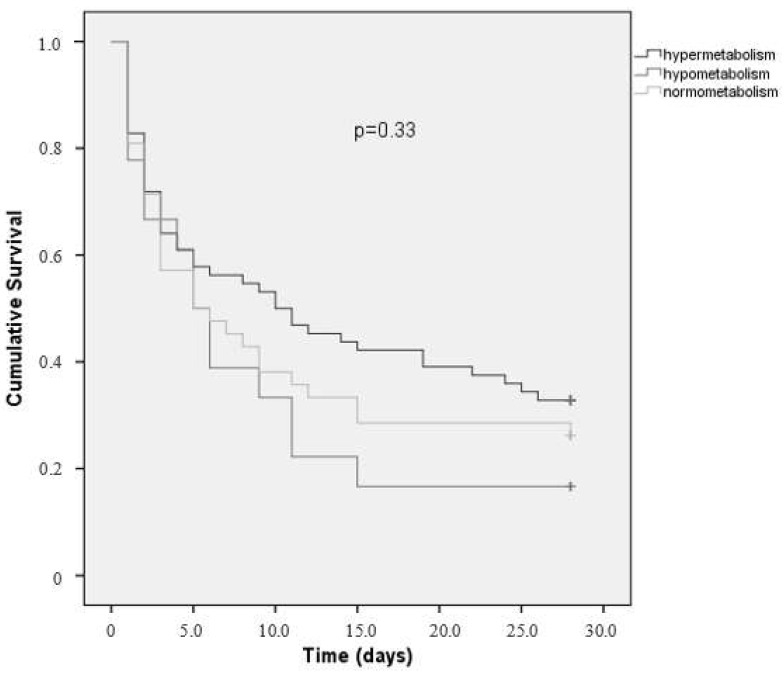
Kaplan–Meier’s 28-day survival analysis for energy expenditure categories.

Analysis of 28-days survival shows that there was no difference in mortality among different REE categories (Figure 1).

## 4. Discussion

In critical patients, important alterations occur in energy and substrate metabolism. Theoretically, critical illness can influence energy use and increase REE significantly [21]. About 35–65% of ICU patients are hypermetabolic [22]. Metabolism categorization has not yet been studied in patients with severe AKI.

There is no consensus in the literature about the diagnosis of hypermetabolism, which makes it difficult to compare studies. Values of REE adequacy greater than 110–115% are used by some authors to diagnose hypermetabolism [18,19]. In our study, patients with REE/BEE >1.3 were diagnosed with hypermetabolism, and using this criteria 62% of patients fit this description. A higher prevalence of this disorder would have been observed in our population if we had used milder parameters for diagnosis. However, as we studied critical patients, we chose a value that would capture patients with a real increase in metabolism, since a lower elevation can be attributed to diet-induced thermogenesis [23,24].

Other studies analyzing different pathologies also observed a high prevalence of hypermetabolic patients [12,18]. Wu et al., assessing septic patients in ICUs, found that 54.8% patients had REE/BEE >1.3 [12]. Less stringent parameters were used by Frankenfield et al. [18] when evaluating critical ICU patients, diagnosing hypermetabolism when REE/BEE >1.15. Probably for that reason, they found a prevalence of 83% of patients with increased metabolism.

In present study, with increasing age, a lower risk of hypermetabolism occurrence was found. Aging is associated with a lower mass of some organs that contribute to energy metabolism [25]. Studies have reported a progressive decline in REE of about 1–2% per decade, and this decline is mostly explained by changes in body composition [26,27,28].

Higher weight was independently associated with the presence of hypometabolism. This association is seen because the Harris–Benedict equation, using real weight, may not be the best tool to estimate BEE in overweight patients [29] as it overestimates BEE, thus categorizing these patients as hypometabolic. As we show, body mass index in the hypometabolic group was higher than in other groups, however, REE measured by IC was significantly lower in the hypometabolic group as compared to the hyper- and normometabolic groups, showing that the energy expenditure in hypometabolic group patients, despite the higher BMI and weight, was actually smaller than in other groups.

The disease and its treatment can greatly alter metabolism and expressively increase EE. The variables that may result in EE increase are pain, fever, and greater severity of inflammatory response [18]. In our study, the hypermetabolic group presented higher body temperature when compared to normometabolic group, but after multiple analysis tests, body temperature did not remain an independent factor associated with hypermetabolism.

Some medications may alter EE in critically ill patients. Sedatives and beta-blockers can reduce EE [30], while the effect of VADs (noradrenaline, for example) on EE has not yet been properly documented. The present study observed that a higher VAD dose was associated with hypermetabolism. Under physiological conditions, metabolic response to catecholamine infusion results mainly in an increased rate of aerobic glycolysis and glucose release from glycogenolysis, neoglycogenesis, and the inhibition of insulin-mediated glycogenesis. In addition to this hyperglycemic response, catecholamines have calorigenic properties, and this metabolic rate increase is associated with greater oxygen consumption, resulting in part from glucose oxidation rate increases [31,32]. Under pathological conditions, metabolic response to catecholamine stimulation is less predictable because of changes in receptor affinity and drug kinetics as a result of disease and treatment used [31].

Although the variables of body temperature, disease severity, and inflammatory response seem to be associated with metabolism, this was not observed in present study. This may be due to the fact that the studied population was homogeneous, with a predominance of septic patients with severe AKI and indications for renal replacement as well as mechanical ventilation. Therefore, clinical variables that are usually associated with hypermetabolism may not have been identified.

Regarding gender, a marginal significance was observed, indicating a possible benefit for males with respect to the occurrence of hypermetabolism. In our study we observed some differences between females and males. For example, men were older (an average age of 61.6 ± 15.4 for men vs. 59.8 ± 19.2, for women), more catabolic (nitrogen balance, in g/d, of −10.5 ± 9 g for male and −0.8 ± 6 for women, *p* < 0.001), and had higher body weight (80.9 ± 23.2 vs. 69.9 ± 10.9, *p* = 0.01). All these clinical parameters were placed in logistic regression adjustment. The only clinical parameter that presented a difference between genders and was not used in adjustment was sedative use, which was higher in men than in women (83.7% vs. 65.8%, *p* = 0.02); this may be one of explanations for this marginal significance observed.

It was expected that hypermetabolism could be associated with a poorer prognosis. However, in present study, there was no association between REE and 28-day survival. Only protein intake and VAD dose were associated with mortality. In the literature, this association is controversial. In critically ill patients with sepsis, Wu et al. [12] observed that the mortality rate in hypermetabolic patients was significantly higher (35% vs. 18%). In the non-surviving group of this study, the energy deficit was intense and difficult to correct due to hemodynamic instability, which may be the cause of the poorer outcome.

As in the Wu et al. study [12], the group who died had lower intakes when compared to the survivor group. However, the hypermetabolic group did not show a greater calorie and protein deficit when compared to other metabolic categories (in contrast, the hypometabolic group presented lower intake), which could explain why we did not find an association between mortality and hypermetabolism.

Calorie and protein intake are known to be important for critically ill patients, and cumulative deficits of both have been associated with increased morbidity and mortality in patients in the ICU [33,34,35,36]. In our study, lower protein intake was independently associated with a higher risk of death (HR = 0.993, 95% CI 0.989–0.999, *p* = 0.032). This result was observed by Allingstrup et al. [35], who evaluated 113 ICU patients and found that higher protein and amino acid sources were associated with lower mortality, and there was no association of energy intake or REE with mortality.

The benefit of protein intake in patients with AKI has been demonstrated in other studies. Kritmetapak et al. [37] studied critically ill AKI patients in continuous renal replacement therapy (CRRT), showed that even after adjustment for severity scores (APACHE and SOFA), serum albumin and creatinine, the daily protein intake remained independently associated with mortality (OR = 4.62, 95% CI 1.48–14.47, *p* = 0.009).

We also evaluated the degree of catabolism in patients through NB, which was negative in all metabolism categories. Hypermetabolic patients were less catabolic than the normal and hypometabolic groups. In this study, calorie and protein intake were only evaluated on the IC measurement day, which may justify the absence of associations between nutritional intake, metabolism, and clinical outcomes.

There are some limitations in this study. Firstly it is an observational cohort study conducted in a single ICU with severe AKI patients, so our results cannot be generalized to all ICU patients.

Second, there are limitations associated with sample size. In addition, a single IC measurement may not characterize EE of these patients and may not reflect the daily variability, which may be significant. Previous studies have shown variability ranging from 16 to 22.6% in EE measured on consecutive days [38,39]. To prevent hyper- or hypoalimentation, IC aiming to guide nutritional therapy should be performed repeatedly. However, in this study, IC was used to assess the metabolism of AKI patients at the time of the indication for dialysis. Another limitation is the absence of body composition assessment. However, precise methods for estimating body composition in critical patients are scarce, and patient transport required for some methods may be associated with complications. Regarding the evaluation of nutritional intake, it is necessary to follow the cumulative deficit to analyze the association between intake and outcomes, which will be done in a future study.

Despite these limitations, this study is the first to evaluate the metabolic categorization of patients with severe AKI and identify the parameters associated with EE.

As a conclusion, patients with severe AKI are mostly hypermetabolic and the presence of hypermetabolism was associated with lower age and higher VAD dose. Hypo- and hypermetabolic patients did not present a greater risk of death in the analysis of 28-day survival, and factors associated with death were low protein intake and higher dose of VAD. More studies are needed to evaluate whether nutritional therapy can influence the outcome of these patients, allowing the association between mortality and energy metabolism categories.

## Figures and Tables

**Table 1:** Clinical characteristics of the general study population.

Parameters (*n* = 124)	
Age (years)	61.08 ± 16.6
Male (%)	86 (69)
AKI etiology (%)	
Associated with sepsis	99 (80)
Ischemia	16 (13)
Nephrotoxicity	7 (5)
Mixed	2 (2)
ATN-ISS	0.65 ± 0.17
BEE (Kcal)	1538 ± 350
REE (Kcal)	2042 ± 703 ^a^
Body Weight (kg)	77.5 ± 22.7
Outcome (%)	
Recovery of renal function	21 (17)
Chronic dialysis	12 (10)
Death	91 (73)

AKI—acute kidney injury, ATN-ISS—Acute Tubular Necrosis Individual Score Severity, BEE—basal energy expenditure, REE—resting energy expenditure. ^a^ *p* < 0.001, when compared to the BEE estimated by the formula.

**Table 2:** Characteristics of severe AKI patients divided by categorization of metabolism.

	Hypermetabolic *n* = 64	Normometabolic *n* = 42	Hypometabolic *n* = 18
Age (years)	59.5 ± 16.5	63.9 ± 17.6	60 ± 14.6
Male (%)	39 (61)	33 (78.6)	14 (78)
ATN-ISS	0.62 ± 0.17	0.68 ± 0.16	0.61 ± 0.23
Serum urea (mg/dL)	177 ± 74	188 ± 84	169 ± 78
Serum creatinine(mg/dL)	4 ± 2.2	5 ± 2	4.5 ± 2.2
CRP (mg/dL)	24.9 ± 13.8	26 ± 15.3	28.5 ± 14.2
TLC (mm^3^)	18,650 (12,300–22,400)	13,500 (11,400–22,300)	19,350 (13,300–27,900)
Body weight (kg)	73.8 ± 19.7	75.3 ± 16	96.2 ± 35.6 ^a^
Body mass index (kg/m^2^)	27.3 ± 7.1	27.2 ± 5.3	33.8 ± 10.3 ^a^
NB (g/day)	−4.45 (−8.4; 1.88) ^b^	−8.4 (−17.8; −4.1)	−6.5 (−22; −2.58)
UNA (g/day)	11.2 (7.01; 15.3) ^b^	18.44 (10.4; 23.9)	11.2 (4.87; 22)
REE (kcal)	2500 ± 616 ^a^	1699 ± 298 ^a^	1214 ± 381.5 ^a^
BEE (kcal)	1464 ± 289	1520 ± 275	1840 ± 523 ^a^
Calorie intake (kcal/day)	580 (0–1500)	550 (0–1250)	0 (0–1050)
Protein intake (g/day)	34.7 (0–85.2)	23.2 (0–76.8)	0 (0–37.5) ^a^
Vm (L/min)	8.8 ± 2.9	8.2 ± 2.2	8 ± 2
Freq (breath/min)	16.8 ± 4.7	15.4 ± 3.3	15 ± 3.8
PEEP	6 ± 2	6 ± 1	7 ± 2
FIO_2_	40 ± 12	38 ± 11	44 ± 12
VAD (mcg/kg/min)	0.28 (0.10–0.77) ^b^	0.12 (0.02–0.23)	0.15 (0.08–0.6)
Sedation use (%)	50 (78)	31 (73.8)	16 (89)
Body temperature (°C)	37.9 ± 1 ^b^	37.5 ± 0.88	37.6 ± 0.93
AKI etiology (%)			
Associated with sepsis	54 (84)	31 (74)	14 (78)
Ischemia	7 (11)	7 (17)	3 (17)
Nephrotoxicity	3 (5)	3 (7)	0
Mixed	0	1 (2)	1 (5)
Primary diagnosis (%):			
CVD	20 (31)	15 (36)	5 (28)
Sepsis	33 (52)	18 (43)	8 (44)
Cancer	5 (8)	3 (7)	1 (5)
Hepatopathy	4 (6)	2 (5)	3 (17)
Trauma	2 (3)	4 (9)	1 (5)
Outcome (%)			
Recovery of renal function	15 (23)	3 (7)	3 (17)
Chronic dialysis	6 (10)	6 (14)	0
Death	43 (67)	33 (79)	15 (83)

AKI—acute kidney injury, ATN-ISS—Acute Tubular Necrosis Individual Score Severity, CRP—C-reactive protein, TLC—total leukocyte count, NB—nitrogen balance, UNA—urea nitrogen appearance, BEE—basal energy expenditure, REE—resting energy expenditure, Vm—minute volume, Freq—respiratory rate, PEEP—positive end-expiratory pressure, FIO_2_—fraction of inspired oxygen, VAD—vasoactive drug, CVD—cardiovascular disease. ^a^ *p* < 0.05, when compared to other categories of metabolism; ^b^ *p* < 0.05, when compared with normometabolic group.

**Table 3:** Multivariate analysis of variables associated with hypermetabolism in severe AKI patients.

	OR	95% CI		*p*
Caloric intake (kcal)	1.000	0.999	1.001	0.563
NB (g/day)	1.035	0.964	1.111	0.345
Creatinine (mg/dL)	0.933	0.696	1.250	0.642
Body weight (kg)	0.983	0.954	1.013	0.271
VAD (mcg/kg/min)	6.493	1.187	35.515	0.031
Body temperature (°C)	0.781	0.411	1.483	0.450
Age (years)	0.960	0.921	0.999	0.046
Gender (Male)	0.299	0.085	1.057	0.061

OR—Odds Ratio, CI—Confidence Intervals, NB—nitrogen balance, VAD—vasoactive drug.

**Table 4:** Multivariate analysis of variables associated with hypometabolism in severe AKI patients.

	OR	95% CI		*p*
Age (years)	0.989	0.928	1.053	0.720
Weight (kg)	0.960	0.923	0.999	0.045
VAD (mcg/kg/min)	0.551	0.093	3.248	0.510
Body temperature (°C)	1.232	0.514	2.957	0.640
Creatinine (mg/dL)	0.963	0.618	1.500	0.867
NB (g/day)	0.961	0.867	1.064	0.443
Calorie intake (kcal)	1.001	0.999	1.002	0.384
Gender (male)	5.210	0.455	59.706	0.185

OR—Odds Ratio, CI—Confidence Intervals, VAD—vasoactive drug, NB—nitrogen balance.

**Table 5:** Characteristics of severe AKI patients according to 28-day survival.

	Survivors *n* = 33	Non-Survivors *n* = 91	*p*
Age (years)	53.73 ± 18.8	63.75 ± 15	0.002
ANT-ISS	0.62 ± 0.16	0.66 ± 0.18	0.18
Body Weight (kg)	74.56 ± 29	78.65 ± 23.7	0.38
REE (kcal)	2216 ± 792	1979.16 ± 661.3	0.09
BEE (kcal)	1516 ± 274	1546 ± 374	0.67
VAD (mcg/kg/min)	0.08 (0–0.2)	0.23 (0.1–0.8)	<0.001
FiO_2_	40.00 ± 11	40 ± 11	0.89
Body temperature (°C)	37.4 ± 0.6	37.8 ± 1	0.04
Serum urea (mg/dL)	170.48 ± 70	183.03 ± 80.4	0.43
Serum creatinine(mg/dL)	5.35 ± 2.8	4.10 ± 1.8	0.004
TLC (mm^3^/dL)	16,350 (11,450; 22,100)	17,900 (12,700–23,900)	0.42
CRP(mg/dL)	24.43 ± 14	26.25 ± 14.34	0.56
NB (g/d)	−6.77 (−14.66; 4.27)	−5.84 (−15; −0.96)	0.58
UNA (g/d)	17 (10; 20.4)	12.6 (7.3; 19.1)	0.33
Calorie intake (kcal)	840 (0–1536)	0 (0–1220)	0.03
Protein intake (g)	64 (0–89.6)	0 (0–73)	0.03
Presence of hypermetabolism			
*n* (%)	21 (63.6)	43 (47.3)	0.24
Presence of hypometabolism			
*n* (%)	3(9.1)	15(16.5)	0.24

AKI—acute kidney injury, ATN-ISS—Acute Tubular Necrosis Individual Score Severity, CRP—C-reactive protein, TLC—total leukocyte count, NB—nitrogen balance, UNA—urea nitrogen appearance, BEE—basal energy expenditure, REE—resting energy expenditure, Vm—minute volume, Freq—respiratory rate, PEEP—positive end-expiratory pressure, FIO_2_—fraction of inspired oxygen, VAD—vasoactive drug.

**Table 6:** Cox regression analysis for association with death.

	HR	95.0% CI		*p*
Age (years)	1.011	0.997	1.025	0.127
REE (kcal)	1.000	0.999	1.000	0.057
VAD (mcg/kg/min)	1.647	1.030	2.634	0.037
Body temperature (°C)	1.317	0.998	1.739	0.052
Creatinine (mg/dL)	0.905	0.809	1.012	0.080
Protein ingestion (g)	0.993	0.987	0.999	0.032

HR—Hazard ratio, CI—Confidence interval, REE—resting energy expenditure, VAD—vasoactive drug.
